# Meta-analysis of reamed versus unreamed intramedullary nailing for open tibial fractures

**DOI:** 10.1186/s13018-014-0074-7

**Published:** 2014-08-23

**Authors:** Yinchu Shao, Hongxing Zou, Shaobo Chen, Jichun Shan

**Affiliations:** 1 Department of Orthopaedics, The 94th Hospital of PLA, Nanchang 330002, Jiangxi, China

**Keywords:** Meta-analysis, Open tibial fractures, Reamed and unreamed intramedullary nailing

## Abstract

**Background:**

Open fractures of the tibial diaphysis are usually caused by high-energy trauma and associated with severe bone and soft tissue injury. Reamed and unreamed intramedullary nailing are often used for treatment of tibial injury. The purpose of this study was to investigate the clinical efficacy of reamed versus unreamed intramedullary nailing for open tibial fractures (OTF).

**Methods:**

A meta-analysis was conducted according to the guidelines of the Cochrane Collaboration using databases containing the Cochrane Library, PubMed, EMbase, Chinese Biomedical Database, Chinese VIP information, and WanFang Database. Randomized and semi-randomized controlled clinical trials of both reamed and unreamed intramedullary nailing for OTF treatment were analyzed using Reviewer Manager (RevMan5.0) software.

**Results:**

A total of 695 references were initially identified from the selected databases. However, only four studies were assessed, matching all the eligibility criteria conducted by two independent reviewers. The result showed that there was no statistical difference in healing rate, secondary surgery rate, implant failure rate, osteofascial compartment syndrome, and infection during the postoperative period between reamed and unreamed nails in OTF.

**Conclusions:**

Findings of this study suggest that there was no statistical difference between reamed and unreamed intramedullary nailing in clinical treatment of OTF. However, the result of this meta-analysis should be cautiously accepted due to some limitations, and further studies are still needed.

## Introduction

Tibial fractures are one of the most common long bone fractures. They can be classified into open and closed tibial fractures according to soft tissue injuries [1]. Open tibial fractures (OTF) are often associated with severe bone and soft tissue injury [2]. Either reamed [3] or unreamed intramedullary nailing technique [4] has been reported to be used in the treatment of OTF. Besides, both of them have different advantages on fracture healing: the reamed nailing has a more rigid structure and earlier fracture union, while the unreamed nailing supplies much better blood to the cortex [5]. However, there is continued controversy regarding the use of reamed and unreamed nailing techniques for the management of OTF [6],[7].

In recent years, systematic review and meta-analysis on the reamed and unreamed intramedullary nailing of some types of fractures have been evaluated. However, the data on the treatment of OTF are insufficient. In this study, a meta-analysis was performed to investigate the difference between reamed and unreamed intramedullary nailing groups for treatment of OTF in terms of postoperative healing rates, secondary operation rates, implant failure rates, osteofascial compartment syndrome, and postoperative infection.

## Materials and methods

### Criteria for selected trials

The inclusion criteria were as follows: (1) published research literature; (2) adult patients with OTF of Gustilo types I, II, IIIA, and IIIB, except for Gustilo type IIIC and old fracture; (3) all randomized controlled clinical trials (RCTs); (4) and the intervention studies on reamed intramedullary nail fixation to patients with OTF in the treatment group and on unreamed intramedullary nailing in patients as a contrast.

Exclusion criteria were as follows: (1) non-randomized controlled trials, (2) observational studies, (3) case reports or review, and (4) the literature research of a sufficient number of patients in treatment and control groups.

### Search methods for identification of studies

Updating to December 2012, the relevant keywords including OTF, intramedullary nailing, and randomized controlled trials were used. The sources of literature search included the Cochrane Library, PubMed, EMbase, Chinese Biomedical Database, Chinese VIP information, and WanFang Database. In addition, we also performed hand searching of information for search strategy.

### Assessment of study quality

The methodological quality of included trials in this study was assessed using the Jadad scale [8]. The evaluation was made according to study design, patients, intervening measure, and observed results. The full texts of all the possibly relevant studies were assessed independently by two reviewers. Any disagreements were resolved by discussion between them or settled by a third reviewer. The Jadad scale score of literatures of more than 3 was considered high quality.

### Data extraction and management

The data were extracted from included reports independently by two reviewers. The data extracted included the following categories: the number of participants, participant characteristics, the study characteristics, risk ratios (RR), mean difference (MD), and the 95 % confidence interval (95 % CI) of the comparisons.

### Assessment of heterogeneity

To determine the heterogeneity of the included studies, the *P* value revealed by the forest plot was used in this study. *I*^2^ was used to estimate the size of the heterogeneity. There was no significant heterogeneity when *P* ≥ 0.05 and analyzed by the fixed effect model. A value of *P* < 0.05 was considered significantly different when analyzed by the random effect model.

### Statistical analysis

All calculations were conducted using Reviewer Manager (RevMan) 5.0 software (Cochrane Collaboration, Oxford, UK).

## Results

### Description of studies

From selected databases, 695 references were obtained. Among them, 21 reviews were excluded from our study. In 674 potentially relevant references, 176 case reports, 243 observational studies, and 241 non-randomized control trials were omitted. The remaining 4 references were taken for a comprehensive evaluation (Table 1). The articles of Keating et al. [9], Finkemeier et al. [10], and Bhandari et al. [11] are in English, while that of Yin et al. [12] is in Chinese. The study characteristics of these 4 studies are shown in Table 1.

**Table 1:** General features of the study

**Study**	**Country**	**Participants (reamed/unreamed)**	**The average follow-up time (months)**	**Jadad grade**
Keating et al. [9]	Canada	50/44	22	2
Finkemeier et al. [10]	USA	25/24	19	3
Yin et al. [12]	China	36/32	18	2
Bhandari et al. [7]	Canada, USA, Holland	221/214	12	5

### The postoperative healing rates in patients with fracture

**Figure 1:**
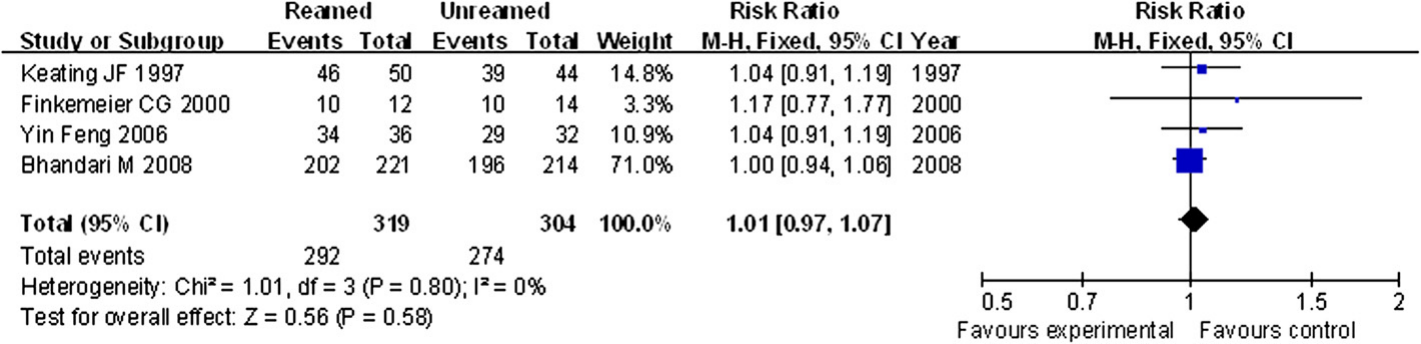
Forest plot of comparison of postoperative healing rates between reamed and unreamed intramedullary nailing for OTF treatment.

All the four studies reported the effect of reamed and unreamed intramedullary nailing on fracture healing rates in patients. Then, a meta-analysis was carried out to compare the healing rates between the two groups. The homogeneity analysis exhibited a good result with heterogeneity test (*P* = 0.80) and *I*^2^ = 0 %. Then, fixed effects model was used for further analysis. The meta-analysis showed that there was no significant difference in postoperative healing rates between reamed and unreamed intramedullary nailing for the treatment of OTF (*P* = 0.58, RR = 1.01, 95 % CI = 0.97–1.07, Figure 1).

### Secondary operation rate in patients

**Figure 2:**
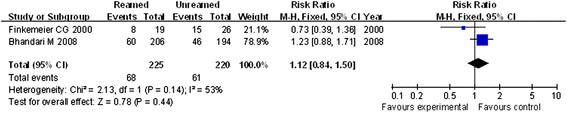
Forest plot of comparison of secondary operation rates between reamed and unreamed intramedullary nailing for OTF treatment.

Finkemeier et al. [10] and Bhandari et al. [11] have reported on the secondary operation rate for patients with fracture. The fixed effect model was used for analysis as a result of the heterogeneity test (*P* = 0.14) and *I*^2^ index (*I*^2^ = 53 %). Then, a meta-analysis was performed, and the result showed that there was no obvious difference in secondary operation rate between reamed and unreamed intramedullary nailing for the treatment of OTF (*P* = 0.44, RR = 1.12, 95 % CI = 0.84–1.50, Figure 2).

### The implant failure rates in postoperative recovery

**Figure 3:**
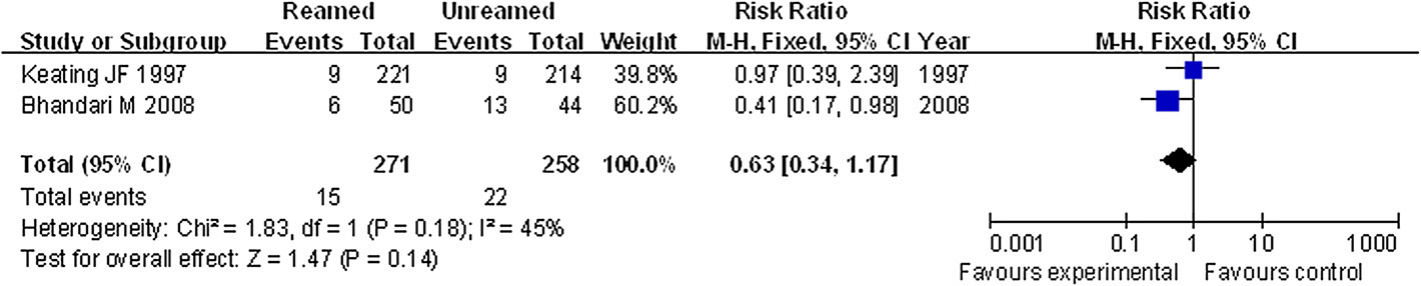
Forest plot of comparison of implant failure rates between reamed and unreamed intramedullary nailing for OTF treatment.

Implant failure events may occur in patients and have been studied by Keating et al. [9] and Bhandari et al. [11] at a stage during the postoperative course. Then, the fixed effect model was carried out based on good homogeneity with heterogeneity test (*P* = 0.18) and index *I*^2^ = 45 %. We observed that the implant failure rates were decreased in patients with reamed intramedullary nail compared to those with the unreamed one. However, there was no significant difference between them (*P* = 0.14, RR = 0.63, 95 % CI = 0.34–1.17, Figure 3).

### The osteofascial compartment syndrome in postoperative recovery

**Figure 4:**
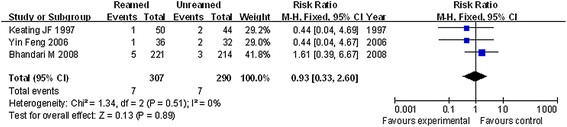
Forest plot of comparison of osteofascial compartment syndrome between reamed and unreamed intramedullary nailing.

According to the good homogeneity with heterogeneity test *P* = 0.51 and index *I*^2^ = 0 %, the fixed effect model was used for further analysis. As shown in Figure 4, there was no obvious difference in osteofascial compartment syndrome between reamed and unreamed intramedullary nailing in patients (*P* = 0.89, RR = 0.93, 95 % CI = 0.33–2.60).

### Postoperative infection

**Figure 5:**
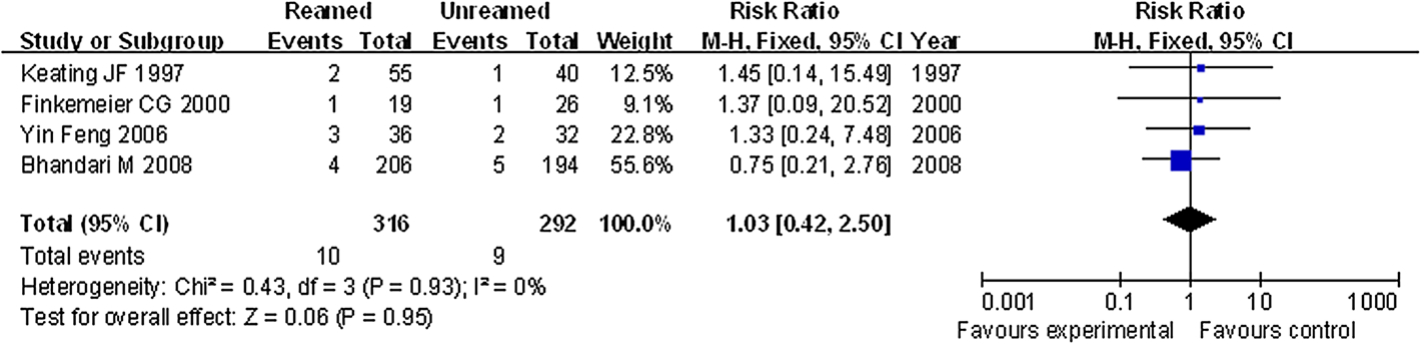
Forest plot of comparison of postoperative infection between reamed and unreamed intramedullary nailing for OTF treatment.

The postoperative infection of patients was reported by all the four references used in this study. The fixed effect model was performed according to the good homogeneity with the heterogeneity test *P* = 0.93 and the index *I*^2^ = 0 %. The results showed that there was no significant difference in postoperative infection of patients between reamed and unreamed intramedullary nailing (*P* = 0.95, RR = 1.03, 95 % CI = 0.42–2.50, Figure 5).

## Discussion

A subgroup analysis of randomized trials of reamed and unreamed intramedullary nailing techniques has been performed for OTF treatment [13]. In this study, both therapies of OTF were evaluated by a meta-analysis. The results showed that there was no difference in the postoperative healing rates, secondary operation, implant failure, osteofascial compartment syndrome, and infection events between reamed and unreamed intramedullary nailing for OTF in postoperative recovery.

Nowadays, there is ongoing controversy about the choice between reamed or unreamed intramedullary nailing approaches for the treatment of OTF. The reamed intramedullary nailing has an advantage in providing optimal biomechanical stability; however, endosteal blood flow damage, bone necrosis, compartment syndrome, and infection may happen due to reaming of the medullary canal [13],[14]. The abovementioned problems associated with reaming do not happen in unreamed intramedullary nailing, but there may be a mechanical stability problem which limits its application [13],[15].

Each surgical option has relative advantages, disadvantages, and indications [16]. It would likewise be attractive to identify patients at risk of developing non-union and to institute procedures for prevention of non-union formation [17]. A previous study suggested that the risk of non-union of femoral diaphyseal fractures could be significantly reduced in reamed intramedullary nailing compared to nonreaming [18]. Taken together, the risk of non-union of OTF may also decrease in reamed intramedullary nailing versus unreamed intramedullary nailing, and the predication requires further investigation. The healing rate of OTF was similar in our study, but it is unclear whether the healing time is different between the two groups. A previous study has found that the average time for tibial fracture healing was 16.7 weeks in the reamed group compared with 25.7 weeks in the unreamed group [19] which indicated that the reamed nailing could shorten the time for healing of tibial fracture.

Anwar et al. [20] showed that surgery of reamed and unreamed intramedullary nailing may lead to pulmonary complications including pneumonia, ARDS, and respiratory failure. It has also been shown that 19.5 % of femur fracture patients who underwent reamed nailing and 9.6 % of femur fracture patients who underwent unreamed nailing had accompanying pulmonary complications, and given the sample size, however, as the inadequate statistical power, definitive conclusions could not be made [20]. In this study, the pulmonary complications of reamed and unreamed intramedullary nailing of OTF are limited and may create directions for future research. There are also other complications in the intraoperative and early and late postoperative periods of OTF treatment, and further studies are still needed.

The economic burden is an important consideration for patients with OTF. One study showed that there was no significant difference in the costs from the index procedure, index hospital stay, and fracture-associated medications; however, the re-operation costs were quite different between reamed and unreamed intramedullary nailing [1]. Our study showed that the re-operation rate was similar in the two treatment groups. This indicated that not only the costs are similar in the two types of surgery but also the re-operation might happen subsequently.

Our findings are mainly limited by the quality and number of included studies. First of all, in terms of the evaluation system, only four studies were incorporated, which might be insufficient for significant effectiveness. Second, there may have been low statistical efficiency in two articles which showed a limited number of patients of no more than 100. Third, other factors including the equipment, medical technology, and judgment index may also influence the evaluation system.

In conclusion, this study showed that there was no significant difference in postoperative healing rates, secondary operation rates, implant failure rates, osteofascial compartment syndrome, and postoperative infection between the reamed and unreamed intramedullary nailing for OTF treatment. However, due to some limitations, the results of this meta-analysis should be cautiously accepted, and long-term follow-up and a larger sample size of high-quality RCTs are needed.

## Competing interests

The authors declare that they have no competing interests.

## Authors' contributions

YS and HZ participated in the design of this study, and they both developed and performed the statistical analysis. SC carried out the study, collected important background information, critically reviewed the study proposal, and drafted the manuscript. JS conceived this study, participated in the design and development of the study, as well as helped to draft the manuscript. All authors read and approved the final manuscript.
